# Functional role of skeletal muscle-derived interleukin-6 and its effects on lipid metabolism

**DOI:** 10.3389/fphys.2023.1110926

**Published:** 2023-07-24

**Authors:** Weimin Lin, Hongbin Song, Jieqiong Shen, Jing Wang, Yue Yang, Yinhua Yang, Jiacheng Cao, Li’e Xue, Fanglu Zhao, Tianfang Xiao, Ruiyi Lin

**Affiliations:** College of Animal Sciences (College of Bee Science), Fujian Agriculture and Forestry University, Fuzhou, China

**Keywords:** skeletal muscle, adipose tisse, myokine, IL-6, lipids metabolism

## Abstract

The detrimental impact of obesity on human health is increasingly evident with the rise in obesity-related diseases. Skeletal muscle, the crucial organ responsible for energy balance metabolism, plays a significant role as a secretory organ by releasing various myokines. Among these myokines, interleukin 6 (IL-6) is closely associated with skeletal muscle contraction. IL-6 triggers the process of lipolysis by mobilizing energy-storing adipose tissue, thereby providing energy for physical exercise. This phenomenon also elucidates the health benefits of regular exercise. However, skeletal muscle and adipose tissue maintain a constant interaction, both directly and indirectly. Direct interaction occurs through the accumulation of excess fat within skeletal muscle, known as ectopic fat deposition. Indirect interaction takes place when adipose tissue is mobilized to supply the energy for skeletal muscle during exercise. Consequently, maintaining a functional balance between skeletal muscle and adipose tissue becomes paramount in regulating energy metabolism and promoting overall health. IL-6, as a representative cytokine, participates in various inflammatory responses, including non-classical inflammatory responses such as adipogenesis. Skeletal muscle influences adipogenesis through paracrine mechanisms, primarily by secreting IL-6. In this research paper, we aim to review the role of skeletal muscle-derived IL-6 in lipid metabolism and other physiological activities, such as insulin resistance and glucose tolerance. By doing so, we provide valuable insights into the regulatory function of skeletal muscle-derived myokines in lipid metabolism.

## 1 Introduction

Currently, the global prevalence of obesity and its associated complications, including cardiovascular and metabolic diseases (Haslam and James, 2005), non-alcoholic fatty liver disease (NAFLD) (Pierantonelli and Svegliati-Baroni, 2019), and type 2 diabetes mellitus (T2DM) (Younossi et al., 2020), continues to rise. These complications pose significant health risks and pose a serious threat to human wellbeing (Cao et al., 2022). Alarmingly, the rates of obesity among children and adolescents have shown a substantial increase from 1975 to 2016 (Lin et al., 2022a). The root causes of obesity can be attributed to the excessive proliferation of adipocytes and the subsequent expansion of adipose tissue (Lin et al., 2022b). With the development of obesity, adipose tissue, particularly subcutaneous adipose tissue, exhibits a remarkable capacity to expand to adapt to energy storage demand requirements through a combination of adipocyte hypertrophy and hyperplasia (Pellegrinelli et al., 2016). Consequently, hyperplastic adipocytes undergo further differentiation and accumulation of lipid droplets, particularly in non-adipose tissue sites, which constitute ectopic lipid deposition (ELD) (Girousse et al., 2019). Skeletal muscle is a common tissue that is widely documented by ELD (Szendroedi and Roden, 2009; de Vries et al., 2014).

The skeletal muscle, as the most critical organ involved in regulating whole-body glucose homeostasis, responds sensitively to insulin (Samuel and Shulman, 2012; Agrawal et al., 2017). It is also an important secretory tissue, which synthesizes and secretes massive myokines involved in various physical activities. Interleukin 6 (IL-6) refers to a potent myokine (Huh, 2018; Severinsen and Pedersen, 2020). It is also a pro-inflammatory cytokine that is secreted by T cells and is considered necessary for the terminal differentiation of B cells (Martínez-Maza and Berek, 1991). With further research, more tissues or organs that can secrete IL-6, including muscles (Febbraio and Pedersen, 2005; Airi et al., 2018; Zhou et al., 2019), have been identified. Skeletal muscle contraction is the primary mode of exercise. According to reports, exercise stimulates IL-6 secretion in the central nervous system and promotes fatty acid oxidation in skeletal muscle by inducing extracellular signal-regulated kinase 1/2 (ERK1/2) phosphorylation (Steensberg et al., 2002; Katashima et al., 2022). Furthermore, the secretion levels of plasma IL-6 increase with skeletal muscle contraction (Keller et al., 2001; Steensberg et al., 2001).

Studies show that IL-6 leads to increased insulin-stimulated glucose disposal uptake, lipolysis, glucose, fatty acid oxidation, and energy expenditure when injected into healthy humans (van Hall et al., 2003; Carey et al., 2006). The physiological role of IL-6 is complex because its characteristics on metabolism require signal integration among different cell types (Scheller et al., 2011; Schmidt-Arras et al., 2016). Moreover, IL-6 has been implicated in promoting increased leptin secretion while suppressing satiety; this interaction promotes adipose tissue lipolysis (Feingold et al., 1992; Pedersen et al., 2003; Wueest and Konrad, 2018). IL-6 increases insulin secretion through an incretin-based mechanism. Indeed, IL-6 tissue-specific knockout mice, including those of the liver, skeletal muscle, and brain, further identify the important role it plays in obesity response (Ellingsgaard et al., 2011; Ferrer et al., 2014; Knudsen et al., 2016; Fernández-Gayol et al., 2019).

Skeletal muscle accounts for 40% of the total body weight; hence, it is the largest organ in the human body, and it acts on health regulation as an endocrine organ (Pedersen et al., 2003). Skeletal muscle also influences the differentiation and proliferation of adipocytes by the myokine through a paracrine mode. Therefore, this review discusses the relationship and function of muscle-derived IL-6 in lipid metabolism, focusing on IL-6 signaling and lipid metabolism in adipose tissue.

## 2 Signaling of IL-6

### 2.1 Classical signaling of IL-6

IL-6 is a phosphorylated glycoprotein consisting of a single chain composed of 184 amino acids. It features four-helix bundles (A-D), with A and B helices running in one direction while C and D helices run in the opposite direction (Kishimoto, 2010; Chen et al., 2022). It is synthesized by fibroblasts, monocytes, macrophages, T cells, endothelial cells, adipocytes, and myoblasts (Mihara et al., 2012).

IL-6 acts on target cells by binding to the interleukin 6 receptor α (IL-6Rα), which is distributed on the surface of the cell membrane. However, it does not signal competence. Initiating signaling requires the association of the IL-6/IL-6Rα complex with glycoprotein 130 (gp130), which is also known as the interleukin-6 receptor subunit (IL-6ST) that acts as the second receptor protein (Kishimoto, 2005). Specifically, IL-6 first binds to IL-6Rα on the surface of the cell membrane, thereby forming a dimer that creates a high affinity for transmembrane gp130, thus aggregating into an IL-6-IL-6Rα-gp130 trimer. Each of the two trimers further forms a homodimer. IL-6 within a single trimer binds to the D1 domain of gp130 within another trimer, thereby further forming a signal-transducing hexameric receptor complex (Boulanger et al., 2003). Notably, prior to the hexamer formation, IL-6 must first be complexed with IL-6Rα, binding to the gp130 receptor for signal transduction (Taga et al., 1989). Subsequently, the trimer activates mitogen-activated protein kinase (MAPK), phosphatidylinositide-3-kinase (PI3K), Janus kinases (JAKs), and signal transducer and activator of transcription (STATs) signaling cascades (Manore et al., 2022). Furthermore, the formation of the IL-6-IL-6Rα-gp130 hexamer recruits the JAK family of non-receptor tyrosine kinases, including JAK1/2 and tyrosine kinase 2 (TYK2), to the cell membrane, which binds to and phosphorylates gp130s cytoplasmic tail at five tyrosine residues (e.g., Y759, Y767, Y814, Y905, and Y915) (Guschin et al., 1995).

**FIGURE 1:**
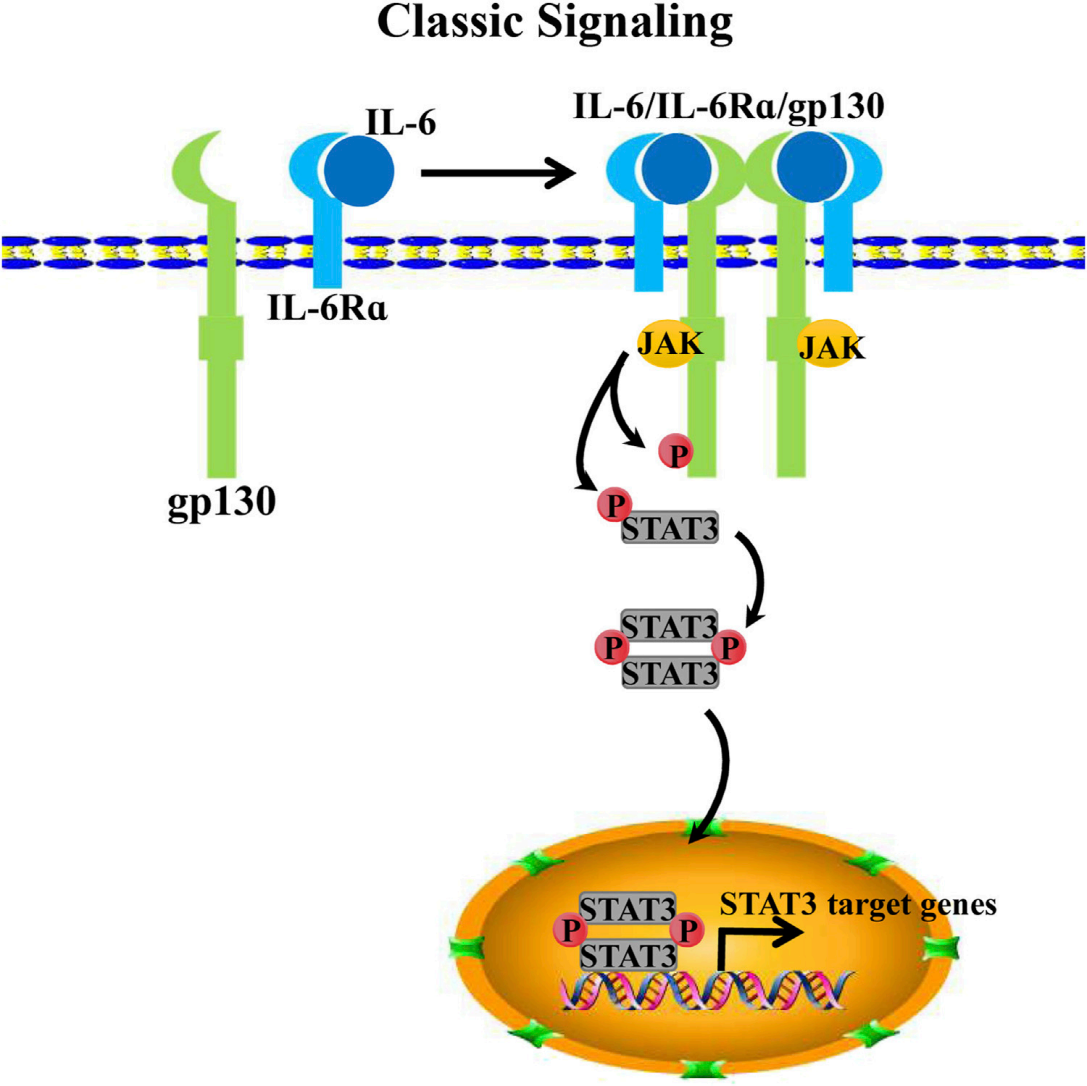
IL-6 classical signaling. IL-6 binds IL-6Rα as a dimeric complex to further form a trimeric receptor complex with pg130. Two IL-6/IL-6Ra/gp130 complexes form a hexameric receptor complex by binding via gp130s D1 domain, thereby activating the intracellular JAK/STAT3 pathway. Recruiting JAKs to the membrane and phosphorylate the cytoplasmic tail of gp130 and STAT3. Phosphorylated STAT3 homodimerize translocate into the nucleus for target transcription activation.

After undergoing phosphorylation, gp130 functions as a docking site for two transcription factors: signal transducer and activator of transcription 1(STAT1) and 3 (STAT3). They are phosphorylated by JAKs at Y701 and Y705 of gp130, respectively (Shuai et al., 1993; Kaptein et al., 1996). Notably, the signaling activation of IL-6/IL-6R/gp130 for STAT3 is more effective than that for STAT1 (Haan et al., 2005). Subsequently, STAT3 is phosphorylated, and its conformation undergoes a change, detaching from the receptor complex and homodimerizing, thereby allowing STAT3 translocation into the nucleus and promoting transcriptional activation for target genes (Morris et al., 2018) (Figure 1).

However, considering that IL-6 has no direct binding capacity to gp130, the expression of IL-6Rα on the cell membrane surface becomes the main limiting factor for IL-6/IL-6Rα/gp130 signaling, which acts as the activation for STAT3 phosphorylation (Taga et al., 1989). Interestingly, the expression of membrane-bound IL-6Rα is restricted to only a few cell types, including immune cells, macrophages, B cells, and subtypes of T cells (Nishimoto and Kishimoto, 2006; Rose-John et al., 2006). Meanwhile, transmembrane gp130 is almost expressed in all cell types (Oberg et al., 2006; Dmitrieva et al., 2016). Considering that IL-6Rα expression is restricted to immune cells and although IL-6 acts with pleiotropic regulatory effects, the non-classical signal outside membrane-bound receptors is subsequently identified as *trans*-signaling (Taga et al., 1989; Müllberg et al., 1993b).

### 2.2 *Trans*-signaling of IL-6

**FIGURE 2:**
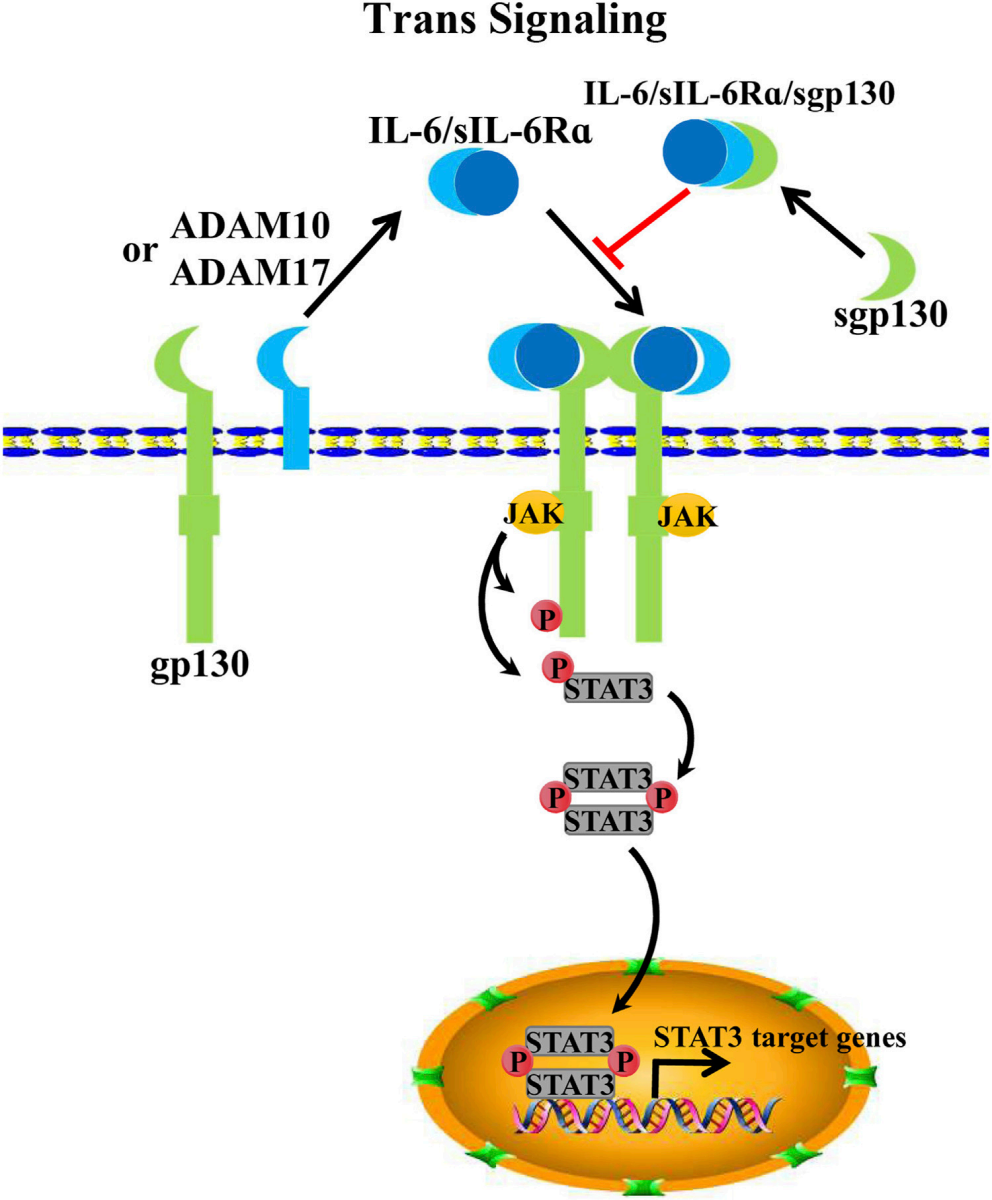
IL-6 *trans*-signaling. Alternative splicing or proteolysis of *IL-6* mRNA by ADAM10/17 can form sIL-6Rα; IL-6 binds sIL-6Rα and forms an IL-6/sIL-6Rα/pg130 hexameric complex for signal transduction to activate JAK/STAT3 signaling. Spg130, on the other hand, usually acts as an antagonist to IL-6/sIL-6Rα, thereby inhibiting IL-6/sIL-6Rα/pg130 signaling.

The key mediator of IL-6 *trans*-signaling is a soluble interleukin 6 receptor α (sIL-6Rα), which potentiates *trans*-signaling in cells that lack sufficient membrane-bound IL-6Rα expression (Taga et al., 1989; Müllberg et al., 1993b). SIL-6Rα is initially detected in the serum and urine of humans, which is considered an agonist for IL-6 signaling. However, with further research, IL-6-sIL-6Rα-gp130 has been identified as a new alternative form of IL-6 signaling (Müllberg et al., 1993b; Toniatti et al., 1996). To date, sIL-6Rα product models include either the proteolytic cleavage of membrane-bound IL-6α or the alternative splicing of *IL-6α* pre-mRNA (Müllberg et al., 1993a; Müller-Newen et al., 1996; Oh et al., 1996). Specifically, upon proteolysis, or ectodomain shedding, membrane-bound IL-6α produces sIL-6α by a disintegrin and metalloproteinase family proteins ADAM10 or ADAM17 (Lust et al., 1992; Garbers et al., 2011; Schumacher et al., 2015). Extracellularly secreted IL-6 dimerizes with sIL-6Rα and then binds to transmembrane gp130 as a trimer. Subsequently, two IL-6-sIL-6Rα-gp130 trimers further homodimerize to activate downstream JAK/STAT3 signaling (Manore et al., 2022) (Figure 2). Notably, gp130 is also present in soluble gp130 form (sgp130). Interestingly, sgp130 acts on IL-6-sIL6Rα dimers as an antagonist to IL-6-sIL-6Rα-gp130, thereby inhibiting IL-6 *trans*-signaling but not impacting IL-6 classical signaling. However, sgp130 is barely expressed compared with sIL-6Rα (Müllberg et al., 1993a; Jostock et al., 2001).

Compared with the classical IL-6/IL-6Rα, which almost only exists in immune cells that mediate the immune response, the *trans*-signaling of IL-6 acts more widely on physiological functions. For immune response, IL-6 *trans*-signaling mediates pro-inflammatory responses by recruiting mononuclear cells, promoting endothelial cells and T-cell survival, and inhibiting T-cell differentiation (McLoughlin et al., 2005; Schaper and Rose-John, 2015). Moreover, IL-6 *trans*-signaling is involved in adipogenesis (Huang et al., 2018), especially in the development of various cancers (Schumacher and Rose-John, 2022). Given that IL-6 *trans*-signaling broadly mediates the pro-inflammatory response, it has been referred to as the main molecular mechanism of IL-6 that acts on tumorigenesis in multiple cancers (McLoughlin et al., 2005; Böttcher et al., 2014).

### 2.3 Cluster signaling of IL-6

**FIGURE 3:**
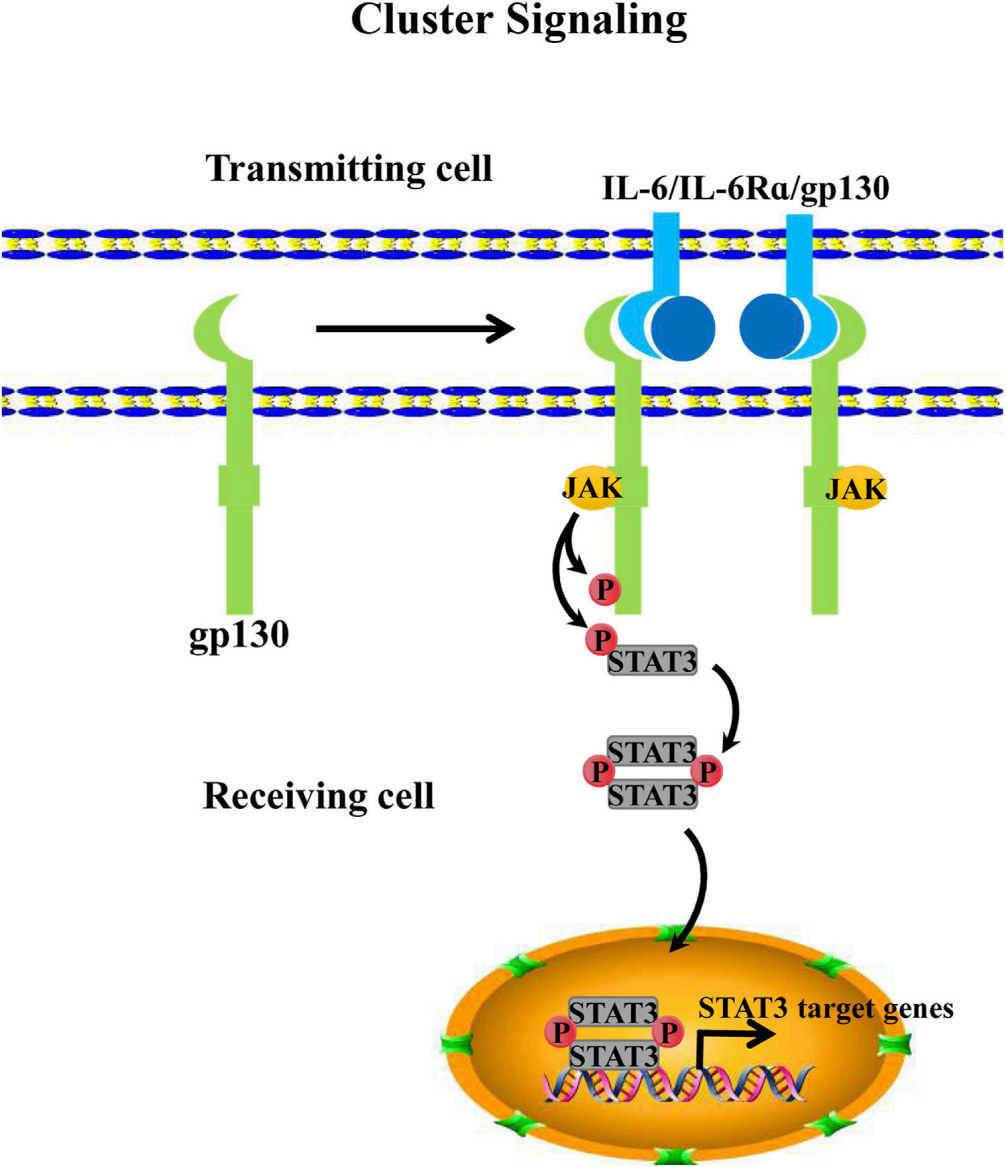
IL-6 cluster signaling. IL-6 cluster signaling consists of 2 cells. The cell that provides IL-6Rα that is binding to IL-6 is usually defined as a “transmitting cell”. Then the dimeric complex forms the trimeric one with pg130 that comes from another cell, this cell is also called the “receiving cell.” Subsequently, the IL-6/IL-6Rα/pg130 complex further activates the intracellular JAK/STAT3 pathway of the “receiving cell”.

In addition to the two aforementioned IL-6 signaling mechanisms, Heink identified a third IL-6 signaling mechanism in 2017. This IL-6 signaling model involves the interaction between two cognate cells, which is referred to as *trans*-presentation or “cluster signaling” (Heink et al., 2017). Specifically, IL-6 dimerizes with the membrane-bound IL-6Rα of dendritic cells (DCs) and then binds to the gp130 receptor of T helper 17 cells (T_H_17). Generally, dendritic cells that provide membrane-bound IL-6Rα are referred to as “donating cells” or “transmitting cells” whereas the T cell that receives the gp130 receptor is generally defined as a “receiving cell” (Chou et al., 2022; Manore et al., 2022). In co-cultured experiments with dendritic cells and T cells, STAT3 signaling activation was in T cells because soluble glycoprotein 130 (sgp130) usually acts as an antagonist to IL-6-IL-6Rα dimer in *trans*-signaling. To investigate whether sgp130 also inhibits “cluster signaling,” Heink et al. detected the function of sgp130 for IL-6-IL-6Rα dimer in cluster signaling. The results showed that sgp130 did not neutralize this signaling model. However, new evidence shows that sgp130 suppresses IL-6 *trans*-presentation signaling by neutralizing the IL-6-IL-6Rα dimer (Bergmann et al., 2017; Lamertz et al., 2018) (Figure 3). Given the contradiction between these findings, the mechanism of IL-6 *trans*-presentation and its biological role remain to be characterized and explored further.

## 3 IL-6 as a myokine

**FIGURE 4:**
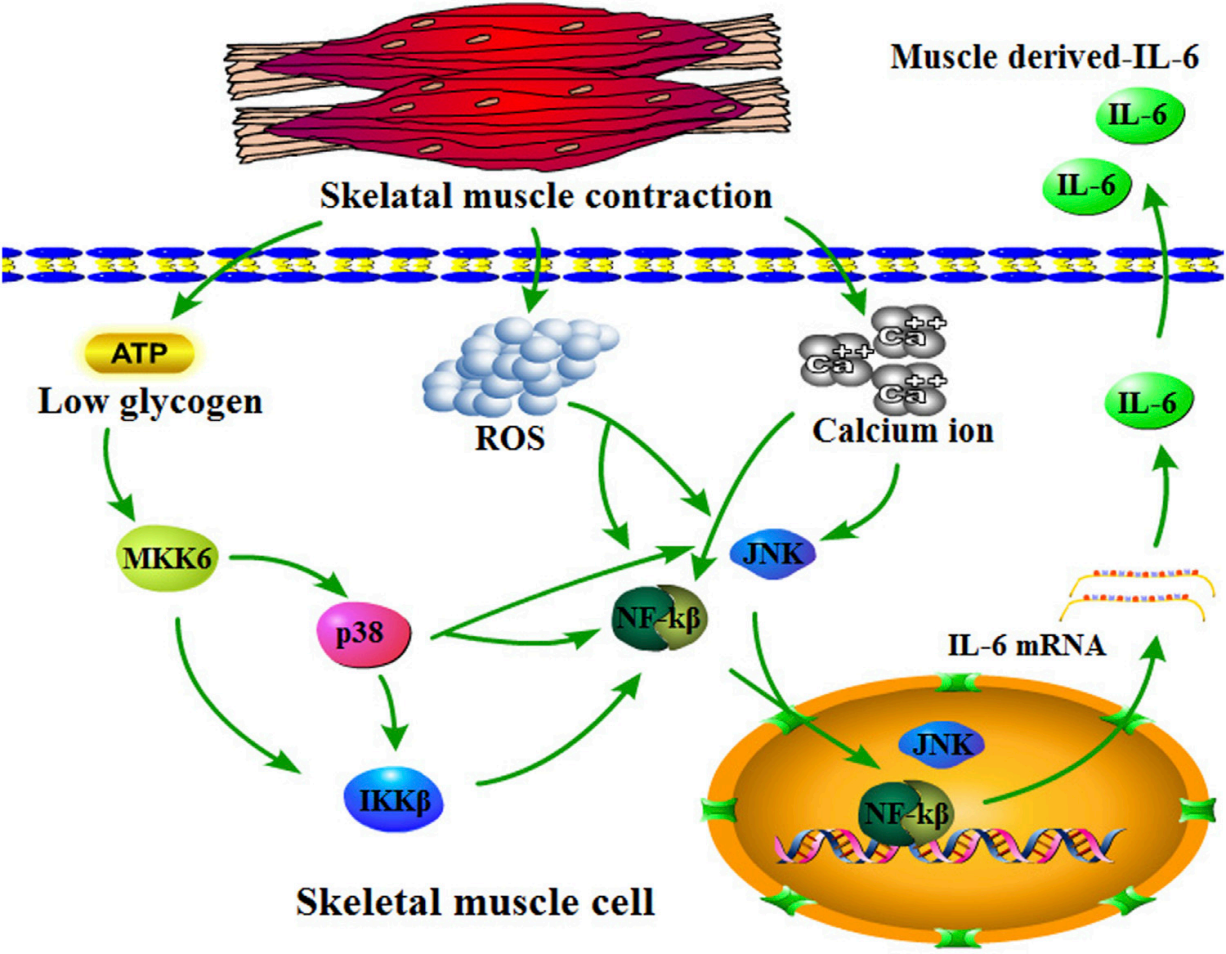
Overview of skeletal muscle-derived IL-6. Skeletal muscle contraction consumes ATP resulting in low glycogen in skeletal muscle cells, which promotes MKK6 to activate p38/IKKβ/NF-kβ or p38/JNK signaling. Moreover, skeletal muscle contraction also upregulates reactive oxygen concentration and calcium ions, which promote NF-kβ and JNK. NF-kβ and JNK translocate into the nucleus for *IL-6* transcription activation.

Skeletal muscle is a crucial organ for maintaining body movement and glucose homeostasis; moreover, it is a secretory organ that acts on multiple physiological activities by the myokines, for example, muscle hypertrophy, fat oxidation, lipolysis, glucose homeostasis, insulin secretion, anti-inflammation, angiogenesis, and bone formation (Pedersen and Febbraio, 2012; Benatti and Pedersen, 2015). Some factors that affect skeletal muscle secretion include diet, exercise, type of myofiber, and genetic factors. For example, a high-fat diet increases the level of saturated fatty acids in the blood, which harms muscle protein synthesis and muscle fiber regeneration while increasing the level of reactive oxygen species (ROS), thereby accelerating proteasome-mediated protein degradation (Woodworth-Hobbs et al., 2014; Brown et al., 2015; Ji, 2015). Exercise remains another factor that affects secreted myokines in skeletal muscles (Sharif et al., 2017; Rosanna, 2019). Among numerous myokines, IL-6 is one of the most strongly associated with exercise. During exercise, serum IL-6 concentrations increased almost 100-fold compared with baseline (Pedersen et al., 2000; Helvoort et al., 2005). Notably, plasma IL-6 levels are associated with exercise duration, training intensity, and the amount of muscle mass mobilized by exercise; specifically, exercise duration is the only determinant of IL-6 release levels. The underlying molecular mechanism of exercise-induced IL-6 secretion by skeletal muscle is the outflow of Ca^2+^ ions. Specifically, Ca^2+^ ions within the sarcoplasmic reticulum liberate the cytoplasm of a skeletal muscle cell during skeletal muscle contraction (Olson and Williams, 2000). More importantly, evidence suggests that Ca^2+^ ion activated nuclear factor kβ (NF-kβ), c-Jun amino-terminal kinase (JNK), and nuclear factor of activated T cells (NFAT) (Dolmetsch et al., 1998). NF-kβ and JNK are activators of the *IL-6* promoter, thereby inducing its transcription (Tuyt et al., 1999). In addition, training intensity involves the depletion of intramuscular glycogen and energy storage. Specifically, the working muscle results in low glycogen (Steensberg et al., 2001; Pedersen and Fischer, 2007; Hojman et al., 2019), which induces p38/MAP kinase (p38/MAPK) to increase, thereby promoting *IL-6* transcription (Boppart et al., 2000; Craig et al., 2000; Chae et al., 2001). Meanwhile, skeletal muscle contraction produces many ROS during exercise. As reported, ROS further induced muscle-derived IL-6 secretion by activating nuclear factor kβ (NF-kβ) signaling, which acts as a transcriptional role for IL-6 (Yamagishi et al., 1997; Bierhaus et al., 2001; Vo et al., 2021) (Figure 4). Exercise-mediated muscle-derived IL-6 is considered the muscle energy sensor that stimulates the release of free fatty acids from lipolysis and adipose tissue, as well as glycogenolysis in the liver that promotes the release of glucose (Pedersen and Febbraio, 2008; Hojman et al., 2019).

Furthermore, muscle-derived IL-6 is released into circulation, acting on other organs or tissues in a hormonal fashion to play various regulatory roles, such as immune response (Fischer, 2007). IL-6 is classified as a pro-inflammatory cytokine. However, with the development of research, IL-6 was shown to be gradually involved in anti-inflammatory effects (Nara and Watanabe, 2021), especially muscle-derived IL-6 (Mauer et al., 2014).

**FIGURE 5:**
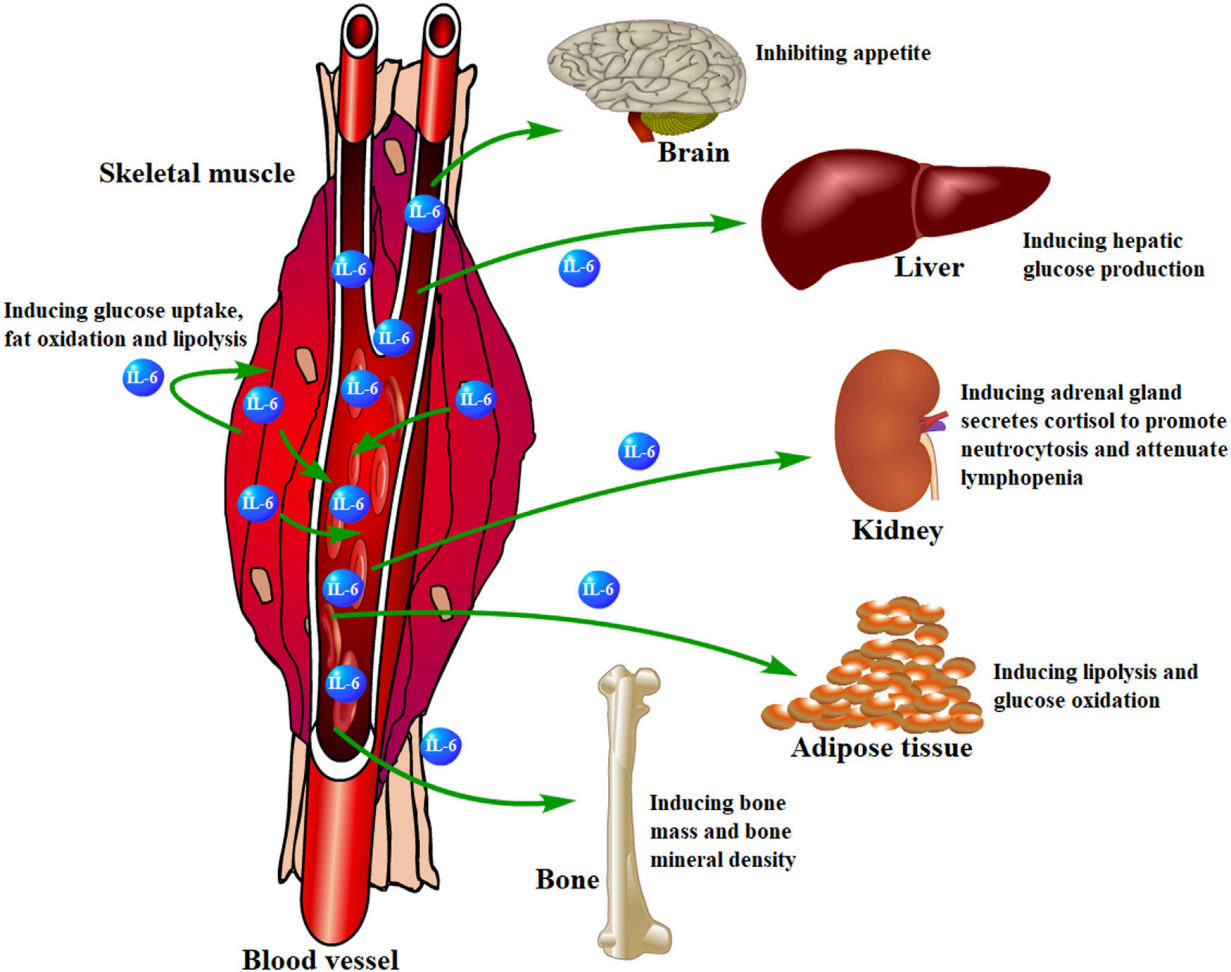
Skeletal muscle-derived IL-6 regulates organs or tissues. Skeletal muscle-derived IL-6 is released into circulation and acts on other organs or tissues in a hormonal fashion to play various regulatory roles.

Besides, muscle-derived IL-6 is involved in regulating physiological activities in other organs or tissues, such as suppressing brain-induced appetite (Schöbitz et al., 1995; Cao et al., 2015), inducing hepatic glucose production (Fritsche et al., 2010; Clementi et al., 2011), glucose oxidation and lipolysis for skeletal muscle and adipose tissue (Hardie et al., 1999; Han et al., 2020), inducing bone mass and mineral density (Barbour et al., 2014; Yokota et al., 2021), and so on (Figure 5).

## 4 IL-6 as an adipokine

**FIGURE 6:**
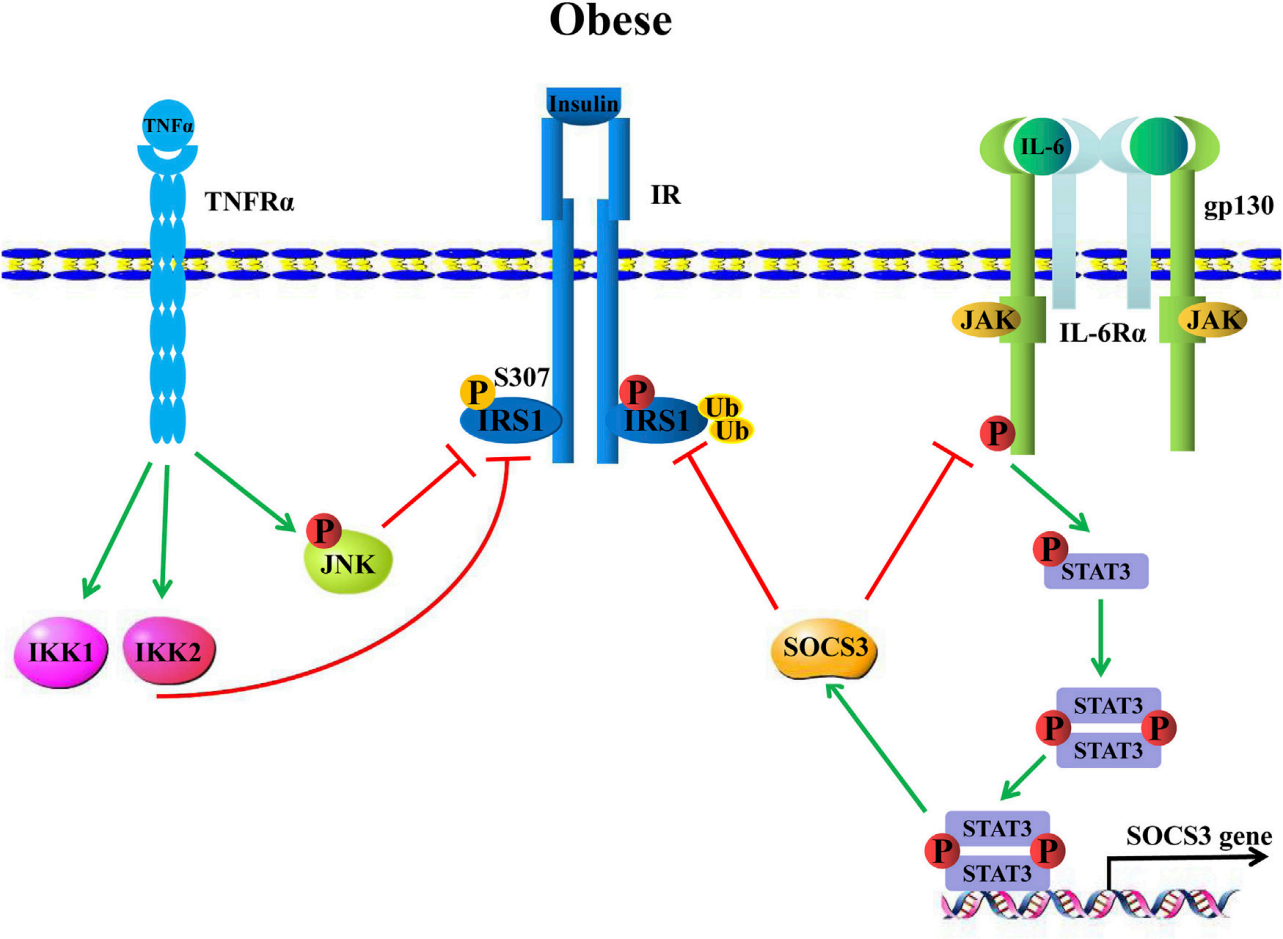
Obesity-induced inflammation causes insulin resistance. TNF-α binds the TNF-α receptor (TNF-Rα) to induce IKK and JNK signals that further attenuate insulin receptor substrate 1, IRS-1 phosphorylation, hence leading to insulin resistance. IL-6, on the other hand, upregulates SOCS3 expression through classical IL-6/JAK/STAT3 signaling, which promotes IRS-1 ubiquitination to induce IRS-1 degradation, thereby inhibiting IRS-1, resulting in insulin resistance.

Adipose tissue, as an important secreted organ that is involved in a series of physiological regulations, is a significant IL-6-secreted organ. With the development of obesity, cell types of innate and adaptive immunity infiltrate obese white adipose tissue (WAT). Among the infiltrating cell types, macrophages are the major subset. Their polarization defines variable functions in obese WATs (Cancello et al., 2006; Murano et al., 2008; Caspar et al., 2009; Lauterbach and Wunderlich, 2017). Of note, adipose-derived TNF-α and IL-6 are crucial adipokines that suppress adipocyte insulin sensitivity and even lead to insulin resistance by attenuating insulin receptor-substrate 1 (IRS-1), which is a necessary component of insulin signaling (Hotamisligil et al., 1996; Rui et al., 2002). For example, TNF-α/tumor necrosis factor receptor 1 (TNFR1)/IRS-1 signaling is one of the most important regulatory axes for insulin resistance. Specifically, TNF-α binds TNFR1 further to activate intracellular c-JUN N terminal kinase (JNK) and IkB kinase (IKK) signals. The phosphorylated JNK (p-JNK) and IKK1/1KK2 further attenuate IRS-1 S307 residue phosphorylation, thereby suppressing insulin signaling activation to induce insulin resistance (Hotamisligil et al., 1996; Peraldi et al., 1996; Liang et al., 2008; Tessaro et al., 2017) (Figure 6). Meanwhile, the signaling axis that IL-6 regulates insulin resistance is considered, IL-6/IL-6Rα/gp130 signaling activates JAK/STAT3 phosphorylation, and p-STAT3 further induces the inhibition of cytokine signaling 3 (*SOCS3*) gene transcription by binding to its promoter, whereas SOCS3 is an inhibitor of IL-6/JAK/STAT3 signaling. Moreover, SOCS3 ubiquitinates IRS-1 to induce its degradation, leading IL-6 to induce insulin resistance (Rui et al., 2002; Sachithanandan et al., 2010; Benito, 2011; Wiejak et al., 2012). (Figure 6) IL-6 secreted from adipose tissue accounts for 15%–35% of the body′s total circulating IL-6 (Mohamed-Ali et al., 1997). More interestingly, IL-6 visceral adipose tissue released three-fold outnumbered subcutaneous adipose tissue (Fried et al., 1998). However, although visceral adipose tissue is the main source of IL-6, subcutaneous adipose tissue-derived IL-6 plays a more important role in regulating glucose metabolism by mediating systemic insulin sensitivity (Bastard et al., 2002).

Moreover, brown adipose tissue (BAT), as non-shivering thermogenesis, is an energy-consuming organ that belongs to another type of adipose tissue (Cannon and Nedergaard, 2004). Generally, BAT is an energy-mobilizing organ that promotes WAT lipolysis and oxidation (Blondin et al., 2014; Garretson et al., 2016; Blondin et al., 2017). BAT adipocytes are more closely related to myogenic cells than WAT adipocytes. BAT adipocytes and myogenic cells originate from Myf5^+^ positive progenitors, and PRDM16 determines myogenic or adipogenic differentiation (Seale et al., 2008; Martinez-Lopez et al., 2013). The structure of BAT adipocytes usually consists of numerous small multilocular lipid droplets and abundant mitochondria. Moreover, BAT adipocytes are smaller in size than WAT adipocytes (Frühbeck et al., 2009). Therefore, BAT acts on thermogenesis that is similar to skeletal muscle rather than WAT. However, BAT still characterizes parts of adipose properties, including inflammatory response (Gallerand et al., 2021), triglyceride storage (Scheja and Heeren, 2016), and secretion effect (Scheele and Wolfrum, 2020). Interestingly, among the types of factors that are BAT-derived or defined as batokines, those that contain IL-6, exploration identifies BAT-derived IL-6 as a necessary factor for improving glucose homeostasis (Stanford et al., 2013; Kalupahana et al., 2020).

## 5 Role of IL-6 in adipogenesis

Given the significant increase in global obesity rates, further exploring the molecular mechanism of adipose tissues is an urgent task for the treatment of obesity-related diseases. Numerous genes or regulators are involved in regulating adipogenesis. Among these genes and regulators, the most critical ones are CCAAT/enhancer binding protein (C/EBP) and peroxisome proliferator-activated receptor (PPAR) families (Lin et al., 2020; Lin et al., 2022a). In addition, other factors are involved in regulating adipogenesis. For example, IL-6 attenuates adipogenesis by lipolysis or fatty acid oxidation. IL-6 adipogenic classical signalings, which typically contain extracellular signal-regulated kinase 1/2 (ERK1/2) signaling, or adenosine monophosphate-activated protein kinase (AMPK) signaling and so on. For instance, IL-6 binds to IL-6R/gp130 and subsequently phosphorylates Raf-1 proto-oncogene, serine/threonine kinase (Raf1), which further induces MAP kinase/ERK kinase 1 and 2 (MEK1/2) phosphorylation, thereby activating ERK1/2 signaling (Ogasawara et al., 2010; Mihara et al., 2012; Latourte et al., 2017). Importantly, in adipocytes, ERK and JNK can phosphorylate peroxisome proliferator-activated receptor γ (PPARγ), thereby attenuating its transcriptional role and further repressing adipogenesis (Hu et al., 1996).

Furthermore, MEK/ERK signaling activation is followed by a decrease in the mRNA levels of phosphorylate peroxisome proliferator-activated receptor γ (*PPARγ*), glucose transporter 4 (*GLUT4*), fatty acid binding protein 4 (*FABP4*), and lipoprotein lipase (*LPL*), which are important adipogenic factors (Brown et al., 2004).

IL-6 also phosphorylates AMP-activated protein kinase (AMPK^Thr172^) via IL-6/IL-6R/gp130 signaling (Kelly et al., 2004; Carey et al., 2006; Khan et al., 2012; Saini et al., 2014; Pacifici et al., 2020). AMPK is known to play a crucial role in lipolysis, which further induces the phosphorylation of acetyl-CoA carboxylase 1 (ACC1^Ser79^) (Luo et al., 2017; Katashima et al., 2022), and ACC1/2 are the limiting enzymes for fatty acid synthesis (Fullerton et al., 2013). AMPK induces phosphorylation to reduce the conversion of acetyl-CoA into malonyl-CoA, which is catalyzed by fatty acid synthase (FASN) to further synthesize the palmitate to form fatty acids so that the phosphorylation of AMPK-induced lipogenesis is attenuated (Saha and Ruderman, 2003; Migita et al., 2009; Day et al., 2017).

AMPK is also involved in increasing Wnt/β-catenin signaling. To put it concretely, AMPK induces the expression of β-catenin and nuclear accumulation in 3T3-L1 cells, which attenuates adipogenic gene expression, including fatty acid binding protein 4 (*FABP4*), CCAAT/enhancer binding protein *α* and β (*C/EBPα* and *C/EBPβ*), Fas cell surface death receptor (*FAS*), phosphorylate peroxisome proliferator-activated receptor γ (*PPARγ*), and sterol-regulatory element binding protein 1c (*SREBP-1c*) (Ducharme and Bickel, 2008; Lee et al., 2011). In addition, adipose triglyceride lipase (ATGL) is a critical triglyceride hydrolase, and AMPK has been demonstrated to mediate ATGL phosphorylation (Ser406), thereby further inducing triglycerides to hydrolyze into fatty acids (Frühbeck et al., 2014; Marzolla et al., 2020; Cho et al., 2021). Peroxisome-proliferator-activated receptor γ co-activator 1α (PGC-1α) has emerged as a master regulator of mitochondrial biogenesis, thus regulating glucose metabolism. AMPK mediates the phosphorylation of PGC-1α (Thr177/Ser538), which is required for PGC-1α-dependent induction of the PGC-1α promoter, thus mediating its transcription (Terada and Tabata, 2004; Lee et al., 2006; Jäger et al., 2007; Ramirez Reyes et al., 2021).

## 6 Role of IL-6 in lipid metabolic diseases

The greatest harm caused by obesity to humans is a series of metabolic diseases that extend from it. These diseases include but are not limited to cardiovascular and metabolic diseases, non-alcoholic fatty liver disease (NAFLD), and type Ⅱ diabetes mellitus (T2DM). Cardiovascular diseases, such as atherosclerotic disease, are primarily caused by lipid deposition in the blood vessel system, which is induced by high-fat food. Gradually, inflammation contributes to atherosclerosis pathogenesis (Libby and Hansson, 2019; O'Keefe, 2019; Tardif et al., 2019). Among thousands of cytokines, NOD-, LRR-, and pyrin domain-containing protein 3 (NLRP3), C-reactive protein (CRP), IL-1, and IL-6 were involved in atherosclerosis pathogenesis (Ridker, 2016; Lutgens et al., 2019; Ridker, 2019). CANTOS, a canakinumab anti-inflammatory thrombosis outcome study, demonstrated that inhibiting IL-1 and IL-6 can reduce the rates of cardiovascular events, especially IL-6, which is directly related to the risk of the occurrence of atherosclerosis (Ridker et al., 2017; Ridker et al., 2018). In particular, beyond IL-6, circulating sIL-R is associated with vascular events and cardiovascular mortality (Hedman et al., 2007; Ritschel et al., 2016).

Furthermore, non-alcoholic fatty liver disease (NAFLD) is a spectrum of liver disorders that usually consist of benign non-alcoholic fatty liver disease (NAFL) and serious non-alcoholic steatohepatitis (NASH). NAFLD is another type of ectopic fat deposition in the liver. It generally contains more than 5% steatosis hepatocytes (Sanyal et al., 2011; Cobbina and Akhlaghi, 2017). Generally, steatosis induces the activation of IKKβ/NF-kβ signaling, thereby promoting a series of pro-inflammatory mediators, including TNF-α, IL-1, and IL-6. They further induce the recruitment and activation of Kupffer cells to mediate inflammation in NASH (Ramadori and Armbrust, 2001; Joshi-Barve et al., 2007; Anderson and Borlak, 2008).

Figure 6 shows that IL-6 leads to insulin resistance, which is one of the most classic features of T2DM, caused by JAK/STAT3 signaling interfering with IRS-1 signaling. Therefore, plasma IL-6 level is one of the biomarkers of T2DM. The report shows that plasma IL-6 levels in T2DM patients are three times that of non-T2DM patients (Skuratovskaia et al., 2021). In obese mice, the plasma IL-6 levels were several fold times higher than those in lean mice (Li et al., 2010).

In summary, IL-6, as an important regulator, is involved in the regulatory process of various lipid metabolic diseases, implying that IL-6 acts as a potential therapeutic target that further plays an important medical role in the subsequent treatment of related diseases.

## 7 Conclusion

Obesity is associated with various metabolic diseases, such as T2DM, which is seriously harmful to human health. For T2DM, the occurrence of ectopic fat deposition in the skeletal muscle is closely related. However, the skeletal muscle serves not only as a vital organ that regulates the body′s energy metabolism but also as an important secret organ that mediates the synthesis and secretion of many myokines. Among these myokines, IL-6 is the most closely related to muscle contraction and it has been proven to mediate lipolysis. Understanding the roles of skeletal muscle-derived IL-6 in lipid mechanisms and other physiological activities is essential in comprehending the interaction between skeletal muscle and adipose tissue through muscle paracrine signaling. This understanding is especially valuable for further exploring the molecular mechanism underlying intramuscular fat deposition and for providing insights into the treatment of diseases associated with ectopic fat deposition.
